# The Effects of School Climate, Parent–Child Closeness, and Peer Relations on the Problematic Internet Use of Chinese Adolescents: Testing the Mediating Role of Self-Esteem and Depression

**DOI:** 10.3390/ijerph19137583

**Published:** 2022-06-21

**Authors:** Hua Wang

**Affiliations:** School of Sociology and Population Studies, Nanjing University of Posts and Telecommunications, Nanjing 210023, China; wanghua@njupt.edu.cn; Tel.: +86-13913037273

**Keywords:** school climate, parent–child closeness, peer relations, problematic internet use, adolescents

## Abstract

Although previous research has investigated the associations among family factors, school factors, peer factors, and problematic Internet use, its causal direction has not been verified, particularly in the Chinese context. Using school-based data, this study aims to explore the possible causal direction among school climate, parent–child closeness, peer relations, and the problematic Internet use of Chinese adolescents. Nine hundred and sixty students in junior and senior high schools participated in a questionnaire survey. The results showed that parent–child closeness, school climate, and peer relations had a significantly direct effect on the problematic Internet use of Chinese adolescents. Meanwhile, the effects of parent–child closeness, school climate, and peer relations on problematic Internet use were mediated by self-esteem and depression. Implications are also discussed to prevent the problematic Internet use of adolescents.

## 1. Introduction

An increasing number of people use the Internet for daily work, socialization, and entertainment in the digital era. At the end of 2021, the worldwide Internet penetration rate is 59.5%, and Internet users have reached approximately 4.66 billion worldwide, up by 7.3% from the previous year [1]. In China, according to the China Internet Development Report (2021), the Internet penetration rate has reached 70.4%, and the number of Internet users reached 989 million by the end of 2020. Among them are approximately 180 million youngsters under 18 years old, accounting for 18.3% of total Internet users [2]. These data show that the Internet has become an indispensable tool for Chinese adolescents. However, compared with the overall global rate of 6%, the prevalence of Internet addiction among Chinese adolescents is approximately 10.4%, which is much higher than that of adolescents in other countries [3]. Problematic Internet use has been labeled as computer addiction, Internet addiction, pathological Internet use, and Internet dependence in previous research. Problematic Internet use was defined as “use of the Internet that creates psychological, social, school and/or work difficulties in an individual’s life” [4]. Previous studies have suggested that generalized and compulsive Internet use leads to loss of control and other negative consequences, such as dissatisfactory school performance or physical and psychological problems for adolescents [5]. With this background, this study aims to investigate the influencing factors and mechanisms of problematic Internet use among adolescents in Mainland China. Empirical evidence is provided for social policy, psychological counseling, and social work interventions to promote the physical and the psychological development of Chinese adolescents.

## 2. Literature Review

### 2.1. School Climate, Parent–Child Closeness, and Peer Relations on Problematic Internet Use

#### 2.1.1. School Climate and Problematic Internet Use

The school climate covers multidimensional factors, such as school values, school culture, teaching quality, interpersonal relationships, and school safety [6,7]. A positive school climate can provide adolescents with a safe, cohesive, and harmonious environment, which has a positive effect on adolescents’ academic outcomes [8], sense of school belonging [9], pro-social behaviors [10], and moral identity [11]. Conversely, a poor school climate may exert negative influences on adolescents’ behavioral and mental development, such as school bullying, problematic Internet use, and depression [12]. Authoritative school climate theory suggests that strict school rules and an emphasis on academics can foster discipline and learning habits, which can prevent students from engaging in unhealthy behaviors [13]. From the development perspective, the positive youth development model suggests that school climate has a positive impact on adolescents’ personality, potential, and self-awareness [14], as well as reducing the probability of adolescents’ problematic Internet use [15,16]. Several empirical studies show that a positive school climate can promote corrective Internet use among adolescents [17]. Conversely, a negative school climate may lead to problematic Internet use [18], including cyberbullying [19] and gaming addiction [20]. Moreover, school types [21] and stress from the school environment [22] are also significantly and positively related to adolescents’ problematic Internet use.

#### 2.1.2. Parent–Child Closeness and Problematic Internet Use

For adolescents, a high-quality family environment contributes to their healthy development. Parent–child closeness is particularly important for adolescents’ development because in the family context, parents take care of their children for a long time [23]. Parent–child closeness refers to the long-term interaction and connection between parents and their children, covering aspects such as parenting styles, family member relationships, family values, and quality of communication [24,25]. According to attachment theory, high-quality parent–child interactions can positively influence children’s physical, emotional, and social development in [26,27]. Proper ways of communication from parents, including patiently listening to children’s ideas, encouraging children to participate in family discussions, and creating a warm family atmosphere, can reduce their children’s fear of missing out and problematic Internet use [28]. Conversely, parents’ punishment and monitoring inevitably lead to children’s insecurity, social fear, and antisocial behaviors [29,30]. Empirical studies have shown that adolescents’ problematic Internet use is caused by limited communication with parents, limited emotional attachment to parents, and poor parenting quality [31,32]. Low quality parent–child relationships is verified to be a main cause of adolescents’ problematic Internet use. In addition, these negative effects are more pronounced for adolescents with low self-control [33].

#### 2.1.3. Peer Relations and Problematic Internet Use

Adolescence is an important transitional period between childhood and adulthood. Social learning theory is often used to explain the correlations between adolescents’ interactions with peers and their behavioral outcomes [34]. The theory argues that peers serve as one of the most important interaction groups for adolescents. In the Internet era, adolescents also learn, imitate, and construct Internet use behaviors from interactions with their peers. Several empirical studies show that peer relations have significant effects on adolescents’ problematic Internet use [35,36]. In a study comparing high school students, girls were found to have much lower problematic Internet use than boys, and peer relationship is negatively correlated to problematic Internet use [37]. Similarly, insecure peer attachment relationships tend to elicit a high risk of problematic Internet use among adolescents [38].

### 2.2. The Mediating Role of Self-Esteem and Depression

#### 2.2.1. Self-Esteem as a Mediator

Self-esteem is a psychological condition shaped by social experiences, including concepts of self-worth, acceptance, and identity [39]. Individuals with high self-esteem may feel positive and confident about themselves. Conversely, individuals with low self-esteem always feel worthless and insecure, and tend to adopt escapist behaviors, such as indulging in Internet use to pursue psychological comfort and subjective well-being [40,41]. Based on a sample of American schools, findings suggest that a healthy school climate can mitigate the effects of self-esteem on problematic Internet use [42]. Similarly, compared with younger pupils, senior students with more awareness of school climate have more positive and distinct trajectories of self-esteem development [43]. Thus, low self-esteem predicts problematic Internet use [44,45]. In other words, adolescents’ problematic Internet use is often closely related to their self-esteem [46], which can then act as a mediating factor in the relationship between school disconnectedness and Internet addiction. In a study examining the effects of parental and peer relationships on adolescents’ self-esteem, the latter did not mediate the effect of communication with mothers, but it significantly mediated the effect of peer relations on problematic Internet use [47]. In addition, parental attachment, peer attachment, and self-esteem are all significantly and positively correlated [48].

#### 2.2.2. Depression as a Mediator

As a common mental disorder, depression is typically characterized by persistent sadness or negative emotions [49,50], making the individuals lose interest in their daily lives [51]. Moreover, depression can have long-lasting adverse effects on physical and behavioral development, such as sleep disturbances, loss of appetite, and distraction [52,53]. The World Health Organization believes that adolescents need to prevent depression by developing self-capacity, self-confidence, and social relationships [49]. School climate, whether positive or negative, may directly promote or hinder adolescents’ mental health [54]. For specific adolescents, the psychological effect of the school climate may be even more significant. For example, homeless youth in California are found to have a significantly high risk of school victimization and a propensity for depression. By contrast, a positive school climate, particularly teacher encouragement and perceived safety, can be effective in mitigating depression [55]. Moreover, parent–child closeness and peer relations can directly affect adolescents’ psychological states. If adolescents have low quality parent–child relationships and high levels of peer stress, their symptoms of depression increase [56]. Conversely, adolescents with secure attachments to their parents and high levels of peer support show fewer depressive symptoms [57]. Female students with depression also have a high risk of problematic Internet use [58]. However, in Minnesota, the relationship between depression and problematic Internet use for students shows no significant difference [59]. Therefore, more empirical studies are needed to further validate this mechanism.

### 2.3. The Present Study

Based on the theories and the empirical evidence, this study aims to explore the possible causal direction among school climate, parent–child closeness, peer relations, and the problematic Internet use of Chinese adolescents. Meanwhile, it also investigates the mediating role of self-esteem and depression on the above mentioned associations. The proposed hypotheses are as follows:

**Hypothesis** **1** **(H1).** *School climate, parent–child closeness, peer relations are more likely to affect the problematic Internet use of Chinese adolescents.*

**Hypothesis** **2** **(H2).** *School climate, parent–child closeness, peer relations would have indirect effects on the problematic Internet use of Chinese adolescents through the mediating role of self-esteem and depression.*

## 3. Method

### 3.1. Participants and Procedures

The data used in this study was obtained by a youth development project in Huai’an in Mainland China. The following are the steps of the multi-stage cluster random sampling method. First, three junior high schools and three senior high schools were selected from a list provided by the educational department using a simple random sample procedure. As a result, six schools were established. Second, three classes were chosen at random from each of the schools. As a consequence, 18 classes was obtained. Finally, 60 students who would take part in the survey were selected using a simple random selection approach. A total of 1080 people were included in the study.

The study adhered to all research ethics guidelines. We initially asked the selected students if they would be willing to take part in our questionnaire survey, and then we declared the confidentiality and authenticity agreement to them. We requested that they and their guardians sign the consent form if they wished to participate in the study. In the end, 1039 students and their guardians signed the consent form. We handed out 1039 questionnaires, and they returned 1006 with a response rate of 96.8%. After double-checking each questionnaires, we were able to collect 960 questionnaires, with a 95.4 percent effective response rate. Furthermore, the author’s university’s Survey and Behavioral Research Ethics Committee examined and approved all research tools.

### 3.2. Measures

#### 3.2.1. Dependent Variables

Problematic Internet use was measured by the problematic social media use (PSMU) scale adapted from the Bergen Facebook Addiction Scale (BFAS) [60], and it consisted of seven items on a five-point Likert scale, with “never, seldom, sometimes, often, always” recorded as “1, 2, 3, 4, 5” points, respectively. “One example in this scale is “tried to cut down on the use of SM without success”. A higher score indicated a higher level of depression in the subjects. In our research, the Cronbach’s alpha for this scale was 0.818.

#### 3.2.2. Independent Variables

The SCBC [61] was used to assess the school climate, which included readiness to seek help, aggressive attitudes, and bullying and teasing predominance. The responses ranged from “strongly disagree = 1” to “strongly agree = 5” for each of the 20 issues. One example in this scale is “if another student was bullying me, I would tell one of the teachers or staff at school”. In this investigation, the Cronbach’s alpha was 0.882.

The revised family communication pattern instrument [62] was used to assess parent–child closeness. This scale was divided in social and concept orientation. This scale comprised 23 items, 5 points for the Likert scale, and ranged from “agree = 1” to “disagree = 5”. One example in this scale is “my parents often asked my opinion when the family was talking about something”. A higher score indicated a greater degree of parent–child closeness. In our investigation, the Cronbach’s alpha for this scale was 0.906.

The peer relationship scale [63] was used to assess peer relationships. It consisted of 29 components that were divided into four dimensions (intimacy, popularity, trust, insightfulness). A five-point Likert scale was used to respond, ranging from “strongly disagree = 1” to “totally agree = 5”. “When I have an issue, I discuss it with my friends,” for example, is an example item for this scale. A higher score indicated a greater degree of peer relations. In our investigation, the Cronbach’s alpha for this scale was 0.947.

#### 3.2.3. Mediating Variable

Rosenberg’s Children’s Self-Esteem Scale (CSES) [64] was used to assess self-esteem. The CSES was a ten-item questionnaire. Each issue was assessed on a 5-point scale, with 1 being the most strongly disagreed with and 5 being the most strongly agreed with. “I believe I possess a variety of positive attributes,” for example, is one of the scale’s examples. The results are added together to get a total score ranging from 5 to 50, with higher values indicating stronger self-esteem. In our investigation, the Cronbach’s alpha for this scale was 0.896.

Fendrich et al. [65] established the Center for Epidemiologic Studies Depression Scale for Children (CES-D), which was used to assess depression. The CES-D consisted of a 20-item questionnaire. Each item was scored on a 5-point scale, with 1 being the worst and 5 being the best. “I didn’t feel like eating, yet I wasn’t too hungry,” for example, is an example of this scale. The results are added together to get a total score that ranges from 5 to 100, with higher scores indicating more depression. In our investigation, the Cronbach’s alpha for this scale was 0.910.

### 3.3. Data Analysis

The data was analyzed using structural equation modeling with Amos 24.0. The modeling fitness of the structural equation model was evaluated using the three criteria of χ^2^, CFI, and RMSEA in this study. (1) χ^2^. The hypothetical model is well matched to the sample data when the chi-square value is non-significant (*p* > 0.05) [66]. Due to sample size sensitivity, it is fairly uncommon for a well-fit hypothesized model to produce a significant χ^2^ if the sample size is too big [67]. (2) The Comparative Fit Index (CFI), which indicates a strong model fit when the value is more than 0.90 [68]. (3) The Root Mean Square Error of Approximation (RMSEA), with values less than 0.05 indicating a ‘tight match,’ and values between 0.05 and 0.08 indicating a ‘good fit’ [69].

## 4. Results

### 4.1. Descriptive Results

The descriptive statistical results of social demographic variables are presented in Table 1. The factors are mainly related to gender, grade, age, and household registration.

### 4.2. Correlations

Pearson correlations (Table 2) were conducted in SPSS 24.0. In this study, all the variables (school climate, parent–child closeness, peer relations, self-esteem, depression, and problematic Internet use) are correlated with each other at a 0.01 significance level.

### 4.3. Measurement Model

The results show that the measurement model provided good fit indices [χ^2^(42, N = 960) = 259.791, *p* = 0.000, and with CFI = 0.902 > 0.9, RMSEA = 0.055 < 0.08]. The three latent variables of parent–child closeness, school climate, and peer relations in the measurement model are well represented by the observed variables (shown in Table 3).

### 4.4. Structural Model

**Figure 1:**
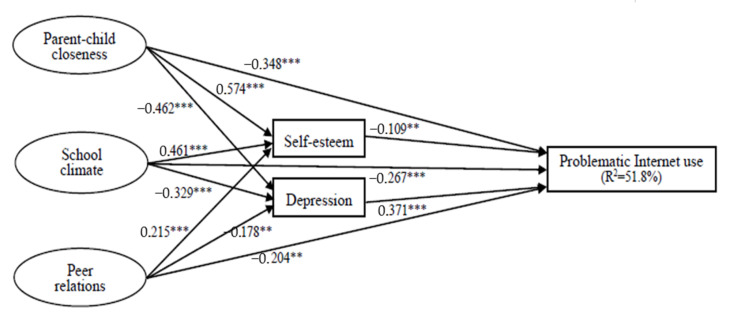
Overall structure equation model (*** *p* < 0.001; ** *p* < 0.01).

Based on the total sample, the results showed a good fit to the data [χ^2^(66, N = 960) = 307.298, *p*= 0.000, and with CFI = 0.917 > 0.9, RMSEA = 0.067 < 0.08]. This suggested that the model was good. Figure 1 demonstrates the paths of this model.

Figure 1 shows that parent–child closeness (β = −0.348, *p* < 0.001), school climate (β = −0.267, *p* < 0.001), and peer relations (β = −0.204, *p* < 0.01) have a significantly direct effect on the problematic Internet use of Chinese adolescents, suggesting that adolescents with higher levels of parent–child closeness, school climate, and peer relations would have lower levels of problematic Internet use behaviors.

Figure 1 also shows that the effects of parent–child closeness, school climate, and peer relations on problematic Internet use are mediated by self-esteem and depression. In terms of the mediator of self-esteem, higher levels of parent–child closeness (β = 0.574, *p* < 0.001), school climate (β = 0.461, *p* < 0.001), and peer relations (β = 0.215, *p* < 0.001) are significantly associated with higher levels of self-esteem, which, in turn, predicts lower levels of problematic Internet use outcomes (β = −0.109, *p* < 0.01). In terms of the mediating effect of depression, higher levels of parent–child closeness (β = −0.462, *p* < 0.001), school climate (β = −0.329, *p* < 0.001), and peer relations (β = −0.178, *p* < 0.01) are significantly associated with lower levels of depression, which, in turn, predicts lower levels of problematic Internet use outcomes (β = 0.371, *p* < 0.01). Together, the overall model accounted for 51.8 percent of the explained variance for adolescents’ problematic Internet use (R^2^ = 0.518).

## 5. Discussion

This study aims to investigate the effects of school climate, parent–child closeness, and peer relations on adolescents’ problematic Internet use. The results show that school climate, parent–child closeness, and peer relations have significant direct effects on adolescents’ problematic Internet use, and this influencing path is mediated through self-esteem and depression. All hypotheses are verified in this study.

### 5.1. The Main Effect Model

This study finds that school climate, parent–child closeness, and peer relations have significant effects on the problematic Internet use of adolescents, which is consistent with previous studies indicating such negative effects on adolescents in the Chinese context [15,16,32,35]. The findings also verify the first hypothesis in our study, which suggests that school climate, parent–child closeness, and peer relations affect the problematic Internet use of Chinese adolescents. In a negative school climate, adolescents without supervision usually neglect discipline, which leads to boredom and self-indulgence. These adolescents often fail to control their behaviors when using the Internet, which in turn creates problematic habits [16,18]. This result is also consistent with the authoritative school climate theory [13]. Given the fierce educational competition among Chinese adolescents, middle schools commonly implement strict discipline to minimize distraction and to keep students focused on their studies. In addition to the effect of school climate, harmonious parent–child relationships can also prevent adolescents’ problematic Internet use. A satisfactory relationship with parents can make adolescents feel safe and give them a sense of belonging, thus reducing the incidence of delinquent behaviors [28]. The finding also illustrates the applicability of attachment theory on the explanation of adolescents’ Internet use in the Chinese context. Adolescents can imitate and influence each other’s behaviors during their interaction with peers, from where—according to social learning theory—adolescents’ problematic Internet use can also emerge [70]. This study supports the view that high quality peer relations can reduce the odds of problematic Internet use, negating the finding that negative peer normative use has no such effect [71]. Therefore, to reduce problematic Internet use among adolescents, policy makers must pay more attention to school climate, parent–child closeness, and peer relations. Conducive measures must be developed and implemented to improve the school climate and to enhance the quality of parent–child relationships. In addition, social workers must make efforts to help adolescents deal with peer relations and attempt to help them to improve their positive attitudes to reduce the risk of problematic Internet use.

### 5.2. The Mediating Model

The results reveal that self-esteem and depression could mediate the effects of school climate, parent–child closeness, and peer relations on adolescents’ problematic Internet use. The findings also verify the second hypothesis in our study, which demonstrates that school climate, parent–child closeness, and peer relations have indirect effects on the problematic Internet use of Chinese adolescents through the mediating role of self-esteem and depression. School climate not only influences adolescents’ learning interest and academic achievement but also their self-esteem [42]. Adolescents with low self-esteem usually lack self-confidence, and they tend to indulge in Internet use [44,72]. Moreover, parent–child closeness can indirectly affect adolescents’ problematic Internet use through the mediating role of self-esteem [73]. Adolescents spend most of their time in the school context and thus peer relations naturally become their most important interpersonal relationship. This study suggests that self-esteem can mediate the association between peer relations and adolescents’ problematic Internet use. Thus, social workers and counselors can help to cultivate adolescents’ self-esteem by improving their self-worth and identity to effectively prevent and to eliminate their problematic Internet use.

The findings suggest that depression can mediate the relationships of school climate, parent–child closeness, and peer relations on problematic Internet use. This finding supports most previous empirical studies [55,56], which indicate the mediating effect of depression. Such negative effects have varying levels of impact on adolescents’ academic achievement and social behaviors [74]. Especially in terms of problematic Internet use, depression shows a significant positive correlation [75], which means that adolescents with depression have a very high risk of problematic Internet use [58]. The results are consistent with a meta-analysis, which confirms the correlation between problematic Internet use and depression in adolescents [76]. Adolescents with depression gradually develop negative attitudes and emotions, and become isolated and disconnected from real life, eventually becoming addicted to the Internet. Thus, from the perspective of social work interventions, the present findings can shed new light on prevention and intervention strategies for adolescents’ problematic Internet use [77], advocating for greater focus on programs targeting depression.

## 6. Limitations

This study also presents certain limitations. First, we investigated the effects of school climate, parent–child closeness, and peer relations on adolescents’ problematic Internet use. The mediating effect of self-esteem and depression was also tested. However, other potential factors (e.g., self-control, personality traits) may affect these mechanisms. Therefore, more comprehensive research is necessary to fill the gaps. Second, this study was carried out with cross-sectional data, which cannot reveal the causal relationship. Thus, longitudinal studies are needed to analyze the direct and the indirect effects among variables in this study. Third, the restrictions of the COVID-2019 caused difficulties in obtaining a nationally representative sample. Thus, data was collected only in Jiangsu province, which limits the generalization of results to a certain extent.

## 7. Conclusions

The prevalence of problematic Internet addiction among adolescents is high all over the world. Based on the authoritative school climate theory, attachment theory, and social learning theory, this study investigated the associations among school climate, parent–child closeness, peer relations, and problematic Internet use as well as the mediating role of self-esteem and depression. Our results showed that parent–child closeness, school climate, and peer relations had a significantly direct effect on the problematic Internet use of Chinese adolescents. Meanwhile, the effects of parent–child closeness, school climate, and peer relations on problematic Internet use were mediated by self-esteem and depression. Based on the findings, we developed an integrated theoretical framework to explain the causal relationship among environmental factors, mental health, and problematic Internet use behaviors. This model can be utilized to explain the influencing mechanism of problematic Internet use behaviors for youth and adolescent groups. What’s more, this study also shed light on the prevention and the intervention programs of adolescents’ problematic Internet use behaviors.

## Figures and Tables

**Table 1:** Sociodemographic characteristics (N = 960).

	Frequency (N)	Percentage (%)
Gender		
Male	474	49.4
Female	486	50.6
Grade		
Junior high school in grade 1	163	17.0
Junior high school in grade 2	171	17.8
Junior high school in grade 3	148	15.4
Senior high school in grade 1	166	17.3
Senior high school in grade 2	157	16.4
Senior high school in grade 3	155	16.1
Household registration		
Rural	84	8.8
Urban	876	91.2
Age	Mean = 14.86	S.D. = 1.639

**Table 2:** Pearson correlations among the key variables.

	1	2	3	4	5	6
1. Parent–child closeness	-					
2. School climate	0.348 **	-				
3. Peer relations	0.256 **	0.331 **	-			
4. Self-esteem	0.339 **	0.323 **	0.443 **	-		
5. Depression	−0.448 **	−0.339 **	−0.440 **	−0.651 **	-	
6. Problematic Internet use	−0.313 **	−0.308 **	−0.187 **	−0.402 **	0.500 **	-

** *p* < 0.01.

**Table 3:** Results of measurement model.

Latent Variables	Observed Variables	β
Parent–child closeness	Social orientation	0.474 ***
	Concept orientation	0.563 ***
School climate	Willingness to seek help	0.612 ***
	Aggressive attitudes	0.567 ***
	Prevalence of bullying	0.688 ***
Peer relation	Intimacy	0.765 ***
	Popularity	0.820 ***
	Trust	0.924 ***
	Insightfulness	0.672 ***

*** *p* < 0.001.

## Data Availability

The data presented in this study are available on request from the corresponding author.
